# Intact Fibrillated 3D-Printed Cellulose Macrofibrils/CaCO_3_ for Controlled Drug Delivery

**DOI:** 10.3390/polym13121912

**Published:** 2021-06-08

**Authors:** Denesh Mohan, Zee Khai Teong, Mohd Shaiful Sajab, Nur Hidayatul Nazirah Kamarudin, Hatika Kaco

**Affiliations:** 1 Research Center for Sustainable Process Technology (CESPRO), Faculty of Engineering and Built Environment, Universiti Kebangsaan Malaysia, Bangi 43600, Selangor, Malaysia; teongzeekhai@gmail.com (Z.K.T.); nhnazirah@ukm.edu.my (N.H.N.K.); 2 Department of Chemical and Process Engineering, Faculty of Engineering and Built Environment, Universiti Kebangsaan Malaysia, Bangi 43600, Selangor, Malaysia; 3 Kolej GENIUS Insan, Universiti Sains Islam Malaysia, Bandar Baru Nilai, Nilai 71800, Negeri Sembilan, Malaysia; hatikakaco@usim.edu.my

**Keywords:** additive manufacturing, bioprinting, fibrillated cellulose, hydrogel 3D printing, polymer composites, drug delivery

## Abstract

The tendency to use cellulose fibrils for direct ink writing (DIW) of three-dimensional (3D) printing has been growing extensively due to their advantageous mechanical properties. However, retaining cellulose in its fibrillated forms after the printing process has always been a challenge. In this study, cellulose macrofibrils (CMFs) from oil palm empty fruit bunch (OPEFB) fibers were partially dissolved for consistent viscosity needed for DIW 3D printing. The printed CMF structure obtained from optimized printing profiles (volumetric flow rate, Q_v_ = 9.58 mm/s; print speed, v = 20 mm/s), exhibited excellent mechanical properties (tensile strength of 66 MPa, Young’s modulus of 2.16 GPa, and elongation of 8.76%). The remarkable structural and morphological effects of the intact cellulose fibrils show a homogeneous distribution with synthesized precipitated calcium carbonate (CaCO_3_) nanoparticles. The shear-aligned CMF/CaCO_3_ printed composite exhibited a sustained therapeutic drug release profile that can reduce rapid release that has adverse effects on healthy cells. In comparison with the initial burst release of 5-fluorouracil (5-FU) by CaCO_3_, the controlled release of 5-fluorouracil can be varied (48 to 75%) with the composition of CMF/CaCO_3_ allowing efficient release over time.

## 1. Introduction

The 3D printing technology has been developing rapidly since its commercialization and extended into various deposition techniques, materials, curing methods, and times [1,2,3]. DIW 3D printing is one of the material extrusion techniques that print liquid-based materials into desired hydrogels for various types of applications [4,5,6,7]. In comparison with other biopolymers, the development of cellulose in the DIW printing method shows great potential in various applications, including medical, electronics, food, and textile applications [3,8]. The first demonstration of cellulose DIW was done by dissolving cellulose in an ionic solution and printing in a nonionic solvent (agar gel), regenerating the cellulose. The printed cellulose model was able to retain its shape until a height of 25 mm, proving the shape fidelity of the printed structure and thus establishing cellulose in various potential printing applications [9]. After that, the cellulose-based DIW was studied with different types of cellulose derivatives, such as cellulose nanofibrils [10,11], cellulose nanocrystals [12,13], regenerated cellulose [14], cellulose acetate [15], carboxymethylcellulose [16,17], and bacterial cellulose [18].

Fibrillated cellulose that is synthesized using high-speed homogenization can be utilized for DIW 3D printing and offers various advantages, such as high surface area, high aspect ratio, and fiber entanglement providing advantageous mechanical properties [19,20]. Currently, fibrillated cellulose printing primarily focuses on using cellulose nanofibrils as they have natural shear-thinning properties that allow smooth extrusion. 3D-printed cellulose nanofibrils can retain their shape after printing due to their high viscosity at the low shear rate [4,21]. However, the more significant challenge is retaining the cellulose in its fibrillated form [22,23]. This limitation decelerates fibrillated cellulose exploitation, which can improve toughness, biodegradability, flexibility, and functionality for nanocomposite fabrication [24]. Besides, cellulose usage in the medical industry has increased tremendously due to its biocompatibility and nontoxicity [25,26,27].

The fibrillated form of cellulose could be used as a binder for the co-drug carrier and therapeutic agents to target and control the release of the drugs (e.g., antibiotics and anticancer drugs) [28]. This is essential to reduce the side effects on the nontargeted cells and tissues during cytotoxic chemotherapy treatment [29]. For example, CaCO_3_ has been used as a drug delivery carrier due to its high availability, biocompatibility, pH sensitivity, osteoconductivity, and slow biodegradability [30,31,32]. It has been used as a drug delivery system for 5-FU, which is utilized in chemotherapy treatment for colorectal, breast, and gastrointestinal cancers [33,34]. However, the loading and release of the therapeutic drug should be controlled to reduce the adverse effects due to the rapid release of the drug into the human body.

In this study, motivated by the cellulose arrangement in lignocellulosic biomass, CMFs were extracted and fibrillated from oil palm biomass. Isolated CMFs were partially dissolved in an alkaline solvent to obtain shear-thinning behavior for DIW extrusion that CMFs do not initially possess. The CMF printing profile with different concentrations was finely optimized with the variation of extrusion flows and printing speeds for the cellulose-printed hydrogel’s accuracy and fidelity. The mechanical properties of the cellulose-based 3D-printed structure depend on the intact cellulose fibrils present through the partial dissolution of CMF. CaCO_3_ encapsulation in the intact cellulose fibril matrix was investigated for controlled therapeutic drug uptake and release.

## 2. Materials and Methods

### 2.1. Materials

OPEFB fibers were procured from Szetech Engineering Sdn Bhd (Selangor, Malaysia) at desired sizes of 106 to 500 µm. The cellulose isolation was done using 95% formic acid, sodium hydroxide (NaOH), hydrogen peroxide (H_2_O_2_), and iron (II) sulfate heptahydrate (Fe_2_SO_4_·7H_2_O) (Merck, Darmstadt, Germany). The tailoring of cellulose rheology was done using lithium hydroxide (LiOH) and urea (Merck, Darmstadt, Germany). The CaCO_3_ nanoparticles were prepared using calcium chloride (CaCl_2_) and sodium carbonate (Na_2_CO_3_) (Merck, Darmstadt, Germany), referring to a previous protocol [5]. Briefly, a 1:1 molar ratio of CaCl_2_ and Na_2_CO_3_ was mixed in deionized water at 200 rpm for 2 h, and the sedimented of CaCO_3_ was washed, centrifuged, and kept dry until further use. 5-FU (Merck) was prepared at 50 ppm in sodium acetate solution (Merck), while phosphate-buffered saline (PBS) (Merck) was used for the drug release medium. 

### 2.2. Cellulose Isolation and Defibrillation

The cellulose isolation from OPEFB fibers was performed by referring to the protocol from previous studies [5,20,35]. The lignin from OPEFB fibers was first isolated using organosolv extraction method using formic acid at a ratio of 30:1 to OPEFB fibers at a temperature of 90 °C for 2 h. The experiment was conducted in a three-necked flat-bottom flask equipped with a condenser. The stirring and temperature were controlled at 800 rpm and 90 °C, respectively, using a digital hotplate magnetic stirrer (MSH-20D, Daihan Scientific, Korea). The supernatant and pulp from the reaction were separated using a vacuum filter (MVP 10, IKA, Staufen, Germany). The pulp was then reacted with NaOH (2 wt.%) and H_2_O_2_ (2 wt.%) to remove hemicellulose and lignin. The cellulosic fraction pulp from the bleaching process was further treated using Fe(II) (10 mg/L) at 90 °C for 24 h for cellulose purification. Continuous washing was done to the cellulose pulp obtained using deionized water, and it was stored in a chiller (4 °C) for further use. The purity of cellulose and lignin content throughout the fractionation process was monitored using National Renewable Energy Laboratory (NREL) standard. 

The concentration of the isolated cellulose was tuned to 2.0 wt.% with deionized water, and it was fibrillated using a high-speed blender (Vitamix 5200, Vitamix, OH, USA) at 37,000 rpm for 10 min to yield CMFs. The temperature during the fibrillation process was kept below 70 °C to prevent cellulose hydrolysis. CMFs were stored in a chiller (4 °C).

### 2.3. Cellulose Macrofibrils Printing Ink

An aqueous LiOH/urea solution with a ratio of 4.6:15 was precooled to −13 °C. Then, isolated CMFs at different concentrations of 3 to 9 wt.% were added into the LiOH/urea solution and vigorously stirred at 3000 rpm for about 15 min before printing to determine the suitable rheology for extrusion and shape fidelity. The solution was frozen again and stirred, with the steps being repeated three times for consistent viscosity. The resulting translucent cellulose solution was centrifuged at 1500 rpm for 10 min to discard the gas bubbles for smooth extrusion prior to printing.

### 2.4. Direct Ink Writing

The prepared CMF solution with different concentrations was fed in a paste extruder to demonstrate CMF solution capability in liquid printing. Discov3ry paste extruder procured from Structur3D Printing, Kitchener, Canada, integrated with Ultimaker 2+ 3D printer (Ultimaker, Utrecht, The Netherlands) was used to print the CMF solution. Computer-aided design (CAD) software (Ultimaker Cura 4.5, Geldermalsen, The Netherlands), according to Discov3ry configuration, was used for selective deposition of CMF solution according to the desired model. The nozzle of 0.84 mm was utilized for all samples. The printing profile of CMFs of different concentrations was optimized by using a 3 cm square of one-layer printing. After the desired samples were printed, the printed structures were regenerated in H_2_SO_4_ (3% *v*/*v*) before further air drying. 

### 2.5. Controlled 5-Fluorouracil Drug Delivery

The 5-FU uptake and release study was performed using the 0.10 g 3D-printed and dried CMF scaffold composited with CaCO_3_ at ratios of 0.25, 0.50, 0.75, and 1.00 with respect to CMF content. The total uptake study was performed by mixing the scaffold in 50 mL 5-FU solution at 150 rpm for 24 h at room temperature. The final solution of 5-FU after centrifugation was analyzed using a UV-Vis spectrophotometer [5,36]. 

The loading efficiency (LE) of 5-FU on the printed structure was calculated using the following equation:

(1) $$\mathrm{LE}(\%)=\frac{C_{0}{-C}_{e}}{C_{0}}\times 100\%$$

where *C*_0_ and *C_e_* are the initial and equilibrium concentrations of the 5-FU (mg/L), respectively. The total 5-FU uptake amount at final equilibrium (*q_e_*) was calculated using the following equation:

(2) $$q_{e}=\frac{{(C}_{0}{-C}_{e})V}{m}$$

where *V* is the volume of the solution (L) and *m* is the mass of the adsorbent (g).

The CMF/CaCO_3_ was then centrifuged at 11,000 rpm for 30 min and dried at 50 °C until constant weight was attained. The release of 5-FU was carried out in PBS solution for 24 h with a small amount of solution being withdrawn hourly for study of the release kinetics. The amount of 5-FU released from the composite scaffold was calculated using the following equation:

(3) $$\;5\text{-}\mathrm{FU}\;\mathrm{release}\;\left(\%\right)=\frac{5\text{-}\mathrm{FU}\;\mathrm{concentration}\;\mathrm{released}\;\left(\frac{\mathrm{mg}}{L}\right)}{5\text{-}\mathrm{FU}\;\mathrm{concentration}\;\mathrm{adsorbed}\;\left(\frac{\mathrm{mg}}{L}\right)}\times 100\%$$

### 2.6. Characterization

The rheological properties of the CMF solution were measured using a Physica MR301 rheometer (Anton Parr) with cone and plate CP25-2 type. The storage modulus (G′) and loss modulus (G″) were measured using oscillatory stress sweep at a frequency of 1 Hz with three repetitions to ensure consistency. The tensile properties of dried 3D-printed samples according to ASTM D638 Type IV were determined using Instron Electromechanical Universal Testing Systems 3300 Series with a load cell of 100 N at a crosshead speed of 1 mm/min (see Figures S1 and S2). The morphological structure of the tensile fractured samples was observed with a field emission scanning electron microscope (FESEM) (Merlin Compact, Zeiss Pvt Ltd., Ober-kochen, Germany), and the element distribution was observed by energy dispersive X-ray analysis (EDX) (Oxford Instruments GmbH, Wiesbaden, Germany). The samples were sputter-coated with gold prior to viewing their morphology. The crystallinity and type of cellulose fractions of the printed samples were determined using an X-ray diffractometer (XRD) (Bruker D8 Advance, Bruker, Billerica, MA, USA) at a diffraction angle from 5 to 60° with a scanning speed of 1°/min. The functional groups of the printed samples were investigated using attenuated total reflectance Fourier transform infrared (ATR-FTIR) spectroscopy (ALPHA FTIR Spectrometer, Bruker, Billerica, MA, USA) in the range of 4000 to 500 cm^−1^ at a resolution of 1 cm^−1^.

### 2.7. Analysis of Variance

The results are expressed in the form of mean ± standard deviation (SD) based on the mean of three replicates (n = 3). The analysis was done with Minitab (statistical software release 18, Minitab, State College, PA, USA). One-way analysis of variance (ANOVA) and Tukey’s test with significance level criteria of *p* < 0.05 were performed using CMF/CaCO_3_ composite printed structures to determine whether all are equal.

## 3. Results

### 3.1. Rheological Patterns of Partially Dissolved CMFs

In the DIW technique, the viscosity of the solution is significant for smooth extrusion and maintaining the shape fidelity of the printed product. The CMF printing ink was prepared by partial dissolution of isolated CMFs in LiOH/urea at concentrations between 3 and 9 wt.%. After dissolution at −13 °C for 15 min, the CMF solution became translucent and showed lesser ability to flow above 4 wt.% cellulose content, indicating the presence of undissolved CMFs. Generally, 3 wt.% isolated cellulose from OPEFB fibers in the urea alkaline system can be dissolved at approximately 90%, depending on the degree of polymerization of cellulose [37]. The dissolved cellulose fraction will help to provide the flowing ability for CMF printing. Meanwhile, at a higher CMF concentration, the undissolved CMF fraction provides the printed structure with shape fidelity and mechanical strength. Hence, the higher concentration of CMFs up to 9 wt.% was utilized for printing for the synergistic effect of partially dissolved CMFs on the printability of CMF solution and shape fidelity of CMF printed structure. A higher concentration than 10 wt.% CMF could not be printed smoothly due to blockage of the extrusion nozzle caused by the high percentage of undissolved CMFs.

**Figure 1:**
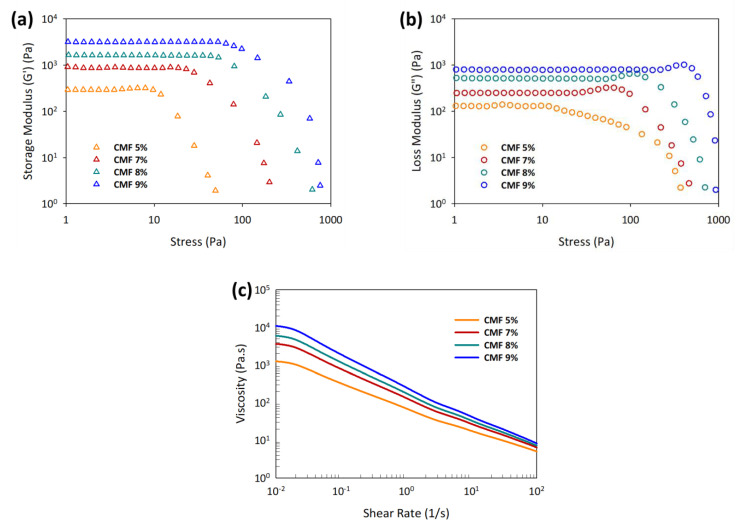
The effect of partially dissolved cellulose on (**a**) storage modulus, (**b**) loss modulus, and (**c**) viscosity at different CMF concentrations.

The rheological pattern of CMF solution at the concentration of 5 to 9 wt.% showed higher storage modulus (G′) than loss modulus (G″) (see Figure 1a,b). This indicates that the printed structure with the CMF concentration from 5 to 9 wt.% will retain its printed shape after printing. Another printing criterion for DIW 3D printing is the yield stress, τ_y_, which is measured at intersection points of G′ and G″ and should be lower than the shear stress on the extruder to ease the extrusion process [14,38]. The yield stress value increased exponentially from 5 to 9 wt.% CMF concentration from 17 to 212 Pa, respectively. The low yield stress indicates that the solution could flow easily, while higher yield stress at high CMF concentrations shows the resistance in flowing caused by the undissolved fraction of cellulose fibers. The yield stress value with respect to CMF concentrations indicates the paste extruder’s specifications to be utilized and the printing parameter optimization. 

CMFs, as they are, have similar characteristics to cellulose in that they do not possess shear-thinning ability when homogenized in water, hence needing a rheological modification step [39]. Importantly, the CMF solution displayed the shear-thinning behavior desired for the DIW printing upon dissolution in LiOH/urea solvent. Figure 1c shows that the viscosity trend has a nonlinear decrement in the log–log plot with the increasing shear rate at different CMF concentrations due to the presence of undissolved CMFs that need higher shear force to break. The increasing CMF concentrations with more undissolved fibers resulted in higher viscosity at a lower shear rate, which helps to retain the shape of the printed structure and desired dimension of the printing with minimal smearing. The viscosity of CMFs at 0.01 s^−1^ drastically increased from 1289 to 11,009 Pa.s at 5 to 9 wt.%, respectively.

### 3.2. Liquid Printing Profile

**Figure 2:**
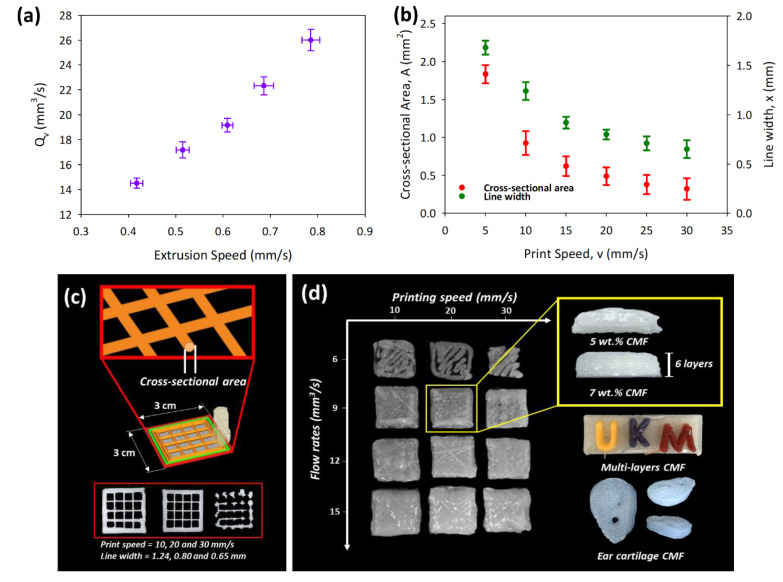
(**a**) CMF solution printing profiles at different extrusion speeds, (**b**) correlation of printing speed with the cross-sectional area and line width, (**c**) the finest printing profiles, and (**d**) different CMF concentrations for multiapplication CMF printing. The data are presented as mean ± standard deviation (n = 3).

The DIW 3D printing of the CMF solution was accomplished using a syringe needle of 0.84 mm internal diameter. The shear-thinning behavior of the CMF printing ink determined in the rheological study enabled the CMF ink to pass the syringe needle with reduced viscosity at a high shear rate and resulted in an increase in its viscosity after printing, allowing the printed shape to be retained. The CMF printing solution was printed as a single line and one-layer geometry to investigate its suitable printing profile; the ambient and build plate temperatures were maintained close to 25 °C to minimize printing dimension variations. The extruder screw speed was varied from 0.4 to 0.9 mm/s to optimize the volumetric flow rate, Q_v_, of the CMF ink through the nozzle (see Table S1). Figure 2a shows the extruder screw speed was kept constant at 0.61 mm/s for subsequent printings, which enabled the extrusion of 9.58 mm^3^/s CMF ink owing to its smooth extrusion.

The printing parameter was further optimized by the correlation of Q_v_; line width, x; printed cross-sectional area, A; and print speed, v. The printing data are tabulated in Table S2 and shown in Figure 2b, which show the decrease of line width with print speed, v, obeying a power-law function, x = 4.1305.v^−0.512^, as related to the principle of conservation of mass [21]. The correlation was done to determine the collapse or expansion of a printed structure at a specific print speed. The print speed was determined to be optimal at 20 mm/s when the printed line width is 0.80 mm, which is close to the nozzle diameter. The mass conservation law, Q_v_ = A.v, was utilized to verify the optimal Q_v_ by determining the printed cross-sectional area, A, multiplied by print speed, v. As shown in Figure 2b, the cross-sectional area was found to be decreasing with print speed and well fitted in the power-law equation, and the printed CMF volumetric flow rate extruded was determined to be 9.56 mm^3^/s, which is close to the desired Q_v_ of 9.58 mm^3^/s. Hence, the printing parameter was optimized with Q_v_ = 9.58 mm^3^/s, v = 20mm/s, and a nozzle size of 0.84 mm supported by accurate printing of square scaffold (see Figure 2c).

The shape fidelity of 3D-printed structures at different CMF concentrations was investigated by printing a cube with the dimensions of 40.32 × 40.32 × 15.12 mm. The viscosity of the CMF solution is vital for the liquid printing profiles, allowing the 7, 8, and 9 wt.% CMF concentrations to be printed close to the designed cube size and retain their shapes without collapsing. Irregularities of the printed structure, such as under-extrusion or over-extrusion, can be observed in Figure 2d when the printing parameters do not follow the optimized printing profile. The printed cube for the lowest concentration, 5 wt.% CMF, shows irregular printed shape compared to digital design and collapsed after the printed structure was kept for 10 min. This is because the storage modulus of 5 wt.% CMF is comparatively closer to the loss modulus, proving poor shape fidelity compared to 7 wt.%. Additionally, multilayer printing of CMF 8% dyed with different colors shows the fidelity of printed letters of “UKM” on the top of the printed CMF solution. The stability of the printed hydrogel without penetration of dyes between printed layers exhibited a consistency of partial dissolution of CMFs for multifunctional cellulose-based printing applications. The fidelity and flexibility of the optimized CMF printing parameters can be further exploited for future 3D bioprinting in tissue engineering, such as printing ear cartilage using CMF 8% as shown in Figure 2d. 

**Figure 3:**
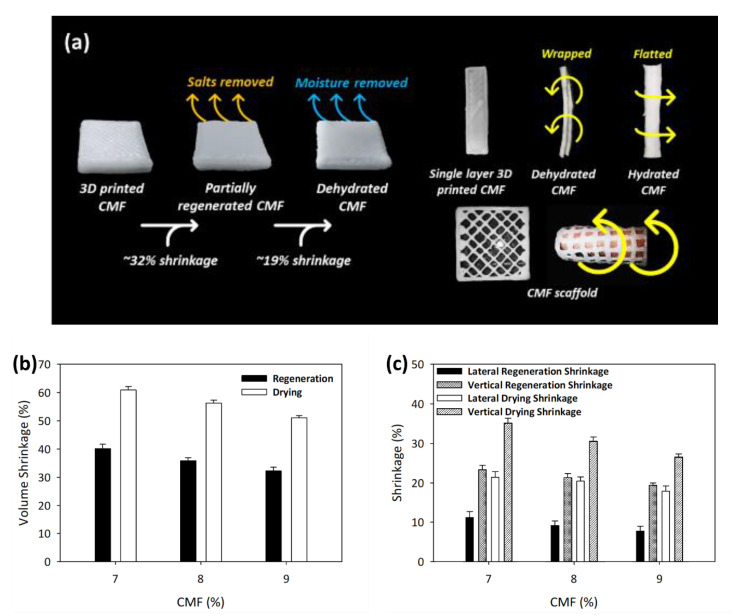
(**a**) Structural changes after dehydration–hydration process, (**b**) total volume, and (**c**) lateral/vertical shrinkages of printed CMFs. The data are presented as mean ± standard deviation (n = 3).

Subsequently, the printed cubes with desired dimensions were regenerated using low concentration acid to remove solvents (e.g., LiOH and urea), thus reducing the translucent appearance of the printed cube [40]. Since water does not dissolve cellulose, cellulose regeneration can be done to the random dissolved cellulose chains that will form an antiparallel cellulose II crystal structure with the alkaline salts being removed [14]. Volume shrinkage was observed with the shrinkage percentage decreasing with increasing CMF concentration for the printed cube during the regeneration and dehydration processes, as shown in Figure 3a. A higher amount of dissolved cellulose will increase the shrinkage, as it will have structural changes to cellulose II with the volume reduction (see Figure 3b). Further drying process increased the shrinkage of printed structures by 19 to 21% due to water removal and formation of hydrogen bonds among CMFs. The shrinkage during regeneration and drying decreases with increasing CMF concentration, which is attributed to the higher undissolved CMF fraction present at higher CMF concentration, which resisted shrinkage, contributing to the improved mechanical properties discussed in the following section.

It was found that the shrinkage of printed cubes was heterogeneous, with more shrinkage in the vertical direction than in the lateral direction, as shown in Figure 3c. This is because the CMFs printed were aligned in the lateral direction, which causes the fibers to become closer during the formation of hydrogen bonds. As the CMF solution was printed layer by layer along the *z*-axis, the vertical shrinkage was found to be higher by around 19 to 23% for regeneration and 26 to 35% for drying, which has been reported for cellulose paper dissolution [14]. The lateral shrinkage was approximately 7 to 11% for regeneration and 17 to 21% for drying, which is less than vertical shrinkage due to resistance to shrinkage by the undissolved fraction of CMFs. Even though the printed structure has heterogeneous shrinkage after regeneration and drying processes, the printed structure can still retain its printed shape and have the high mechanical strength suitable for use in further applications.

Besides the controlled shrinkage of the printed cubes, the single-layer printed structure showed shape-responsive properties in the hydration and dehydration process, as the structure could wrap itself under drying condition and unwrap upon water absorption (see Figure 3a). This is due to the dissociation of hydrogen bonds between cellulose chains during water absorption and hydrogen bond restoration during drying. The incomplete water removal during the regeneration and drying process and controlled water absorption capability of printed CMF structure will increase the tensile strength ascribed to the swelling of the CMFs [41]. The printed CMF structure also showed shape flexibility in that the printed scaffold could be wrapped on a human finger, allowing the application of wound healing to be considered. The potential of CMF printed structures can be further exploited by functionalization with chitosan/alginate for wound dressing and controlled drug delivery applications [42].

### 3.3. Mechanical and Morphological Properties of the Printed CMF

**Figure 4:**
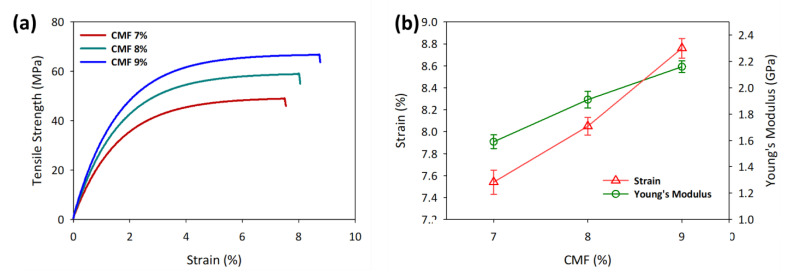
Mechanical properties of printed samples of CMFs at different concentrations in (**a**) stress–strain curve and (**b**) Young’s modulus. The data are presented as mean ± standard deviation (n = 3).

Figure 4 shows the tensile properties of the printed CMF structure according to ASTM D638 Type IV. The tensile strength of 7, 8, and 9 wt.% printed CMF was 48.96, 58.89, and 66.70 MPa, respectively (see Table S3). The high tensile strength is associated with hydrogen bonds between the cellulose chains that were not entirely broken during LiOH/urea dissolution at 9 wt.% CMF concentration has more undissolved cellulose fibers; this is verified by the crystallinity of cellulose I fraction, which is 2.25 times that of cellulose II fraction [43]. The tensile properties of the printed CMF structure are considerably homogeneous due to the breaking at the necking region, as shown in Figure 4a. Interestingly, CMF 9 wt.% tensile strength at 66.70 MPa is indeed higher than commercial printing paper (43.5 MPa) [44], commercial 3D printing ABS (38.0 MPa) [45] and PLA (40.3 MPa) [46] polymer and only slighter lower than cellulose nanofibrils films (72.6 MPa) [21] showing the potential of CMF DIW 3D printing. However, if compared with the tensile strength of cellulose from the casting method, the tensile strength of 3D-printed CMF structure is comparatively lower than the cast films due to bubbles during extrusion and the lack of molding pressure that increases the porosity of the printed samples [44,47,48].

The Young’s modulus of the tensile samples showed a similar trend, where it increases with the increment in CMF concentrations (see Figure 4b). The higher amount of undissolved cellulose fibers at 9 wt.% CMF in fibril form increased the stiffness of the printed sample by 39% compared to that at 7 wt.% CMF. The tensile modulus of 9 wt.% CMF at 2.16 GPa is lower compared to the tensile modulus of a single cellulose fibril, which can be more than 100 GPa [49], due to voids present in the CMF printed structure. However, the modulus can be considered high when compared with commercial 3D printing materials such as ABS (1.71 GPa) [45], PLA (2.86 GPa) [50], and some cellulose-derived films such as 3D-printed cellulose nanofibril films (10.2 GPa) [21], bacterial cellulose (0.3 GPa) [51], and carboxymethyl cellulose (0.02 GPa) [52]. On the other hand, the elongation at break of 9 wt.% CMF is 8.76%, which is the highest and comparable to the cast regenerated cellulose [53]. Overall, the tensile properties increased with CMF concentration, as the undissolved fraction of cellulose fibers increased, thus increasing the hydrogen bonding between the cellulose fibers. With this CMF DIW 3D printing method, a mechanically strong and complex CMF structure can be printed with the aligned fibrillated form of cellulose still intact.

**Figure 5:**
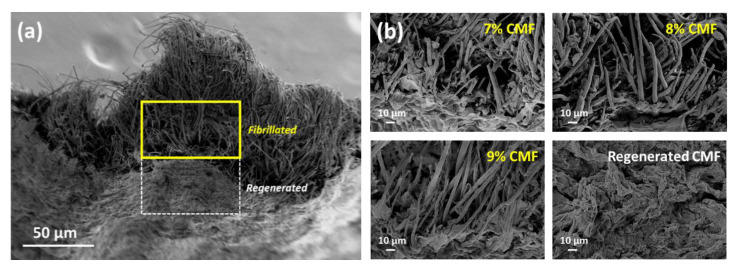
(**a**) Morphological differences of partially regenerated CMFs; (**b**) different cellulose concentrations with fibrillated and regenerated CMF structures.

Figure 5 shows the microscopic morphologies of the 3D-printed and regenerated fractured tensile samples at different CMF concentrations that can be used to study the intact cellulose fibrils and alignment of cellulose fibrils. Notably, the fractured tensile samples at the cross-sectional area have protruding fibers that mimic the real arrangement of cellulose fibrils in plants [54]. The intact cellulose fibrils at CMF concentrations of 7, 8, and 9 wt.% verify the XRD results showing that there is a presence of undissolved cellulose fraction. The high concentration CMF with undissolved cellulose fibers maintains the shape fidelity of the printed structure after the extrusion and regeneration–drying process. Figure 5b portrays microscopic images under higher magnification, showing aligned printed cellulose fibrils; this indicates that the method can be further developed to selectively align cellulose fibers for applications that require high surface area. The fraction of CMFs that dissolved in the alkaline solvent and was regenerated had helped the cellulose fibrils become packed, helping maintain the shape of the printed structure, even in the occurrence of shrinkage. The increment in CMF concentration also shows some decrement with porosity, as the 9 wt.% CMF with protruding cellulose fibrils was seen to be closely packed after regeneration and drying, which provides remarkable mechanical strength to the cellulose-based printed structure.

### 3.4. Chemical Characterization

**Figure 6:**
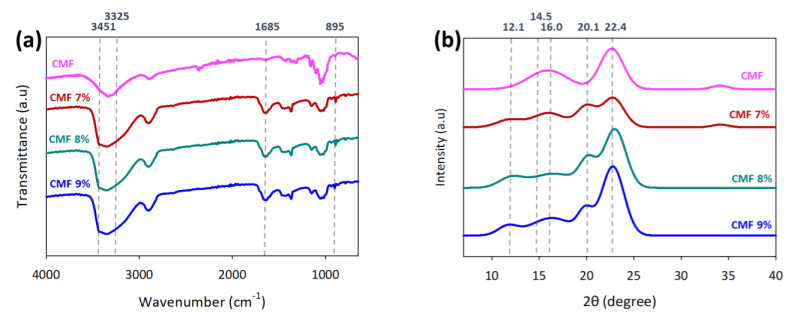
Chemical characterization of neat CMFs and 3D printed CMFs on (**a**) FTIR and (**b**) XRD at different CMF concentrations.

Figure 6a shows the FTIR spectra of neat CMFs and regenerated CMFs. Peaks appear at 3490 and 3445 cm^−1^ after regeneration, representing the stretching of formed hydrogen bonds; the peak at 1685 cm^−1^ is attributed to the C–O carbonyl stretching vibration, and the peak at 895 cm^−1^ is attributed to the glycosidic linkage in the cellulose [55,56]. There is a notable increase in vibrations at 895 cm^−1^, indicating an increment in the amorphous region of cellulose II in the regenerated CMFs compared with CMFs due to some crystallinity properties were compromised during the regeneration process. However, the peak reduced with increasing CMF concentration due to increasing undissolved CMF fraction [57].

XRD pattern shows the crystallinity change during CMF dissolution, as shown in Figure 6b. The neat CMF XRD spectrum displayed cellulose I peaks at 14.5, 16.0, and 22.4°, attributed to the (110), (1Ī0), and (200) planes, respectively [5,58]. The CMF treated with LiOH/urea displayed weakening cellulose I and cellulose II peaks that appeared at 12.1 and 20.1°, indicating the partial change to the cellulose II crystalline state [59]. With increasing CMF concentration for dissolution, cellulose I peaks become more prominent over cellulose II peaks, showing an increment in undissolved cellulose fraction in the LiOH/urea solution. As shown in Table 1, the cellulose I percentage was increased from 8 to 69% and the crystallinity index (CrI) increased from 46 to 61% as CMF concentration increased from 3 to 9 wt.%, respectively. The fractions were expected owing to the partial dissolution of CMFs and intact fibrillated cellulose fibers after printing and regeneration processes.

### 3.5. Controlled Release of 5-FU Therapeutic Drug

**Figure 7:**
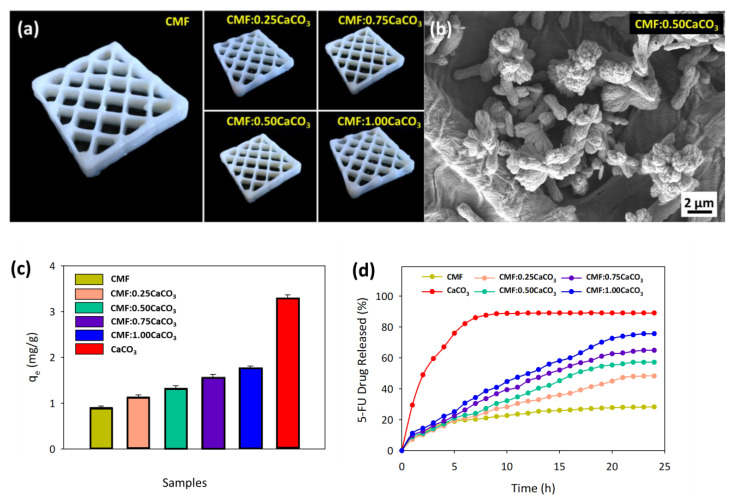
(**a**) Printed CMF/CaCO_3_ scaffold, (**b**) morphological view of CMF/0.50CaCO_3_, (**c**) adsorption capacity of CMF/CaCO_3_ composite, and (**d**) 5-fluorouracil drug release profile. The data are presented as mean ± standard deviation (n = 3).

CaCO_3_ was chosen to be composited with CMFs in this study as CaCO_3_ can rapidly adsorb and release a drug, while nanocellulose can control the drug release percentage, according to previous findings [5,60]. The shape fidelity of the nanocellulose–CaCO_3_ composite was low as the drying process eliminated most of the water content. This can be addressed using partially dissolved CMFs as they have only around 50% shrinkage, and the printed CMF/CaCO_3_ with different ratios of CaCO_3_ can retain the printed scaffold shape, as shown in Figure 7a. As observed in Figure 7b, good dispersion of CaCO_3_ on the CMFs can be seen without significantly affecting the printing parameters and printed composite structure. Accordingly, ~28.4% of Ca was present in the mixture of CMF/CaCO_3_ in the EDX analysis, as shown in Figure 7b.

The absorption spectrum of 5-FU was recorded from 250 to 400 nm. The wavelength to determine 5-FU concentration was determined to be 266 nm for 5-FU concentration of 1–10 ppm [5]. The standard linear curve equation was calculated to be y = 0.0606x + 0.0172 (see Figure S3) with a high regression value of R^2^ = 0.99, indicating a good correlation between theoretical and experimental values. The 5-FU uptake study was carried out using the water-regenerated and dried CMF/CaCO_3_ composite printed scaffold at 8 ppm 5-FU concentration. The LE and adsorption capacity found for CaCO_3_ were 82.49% and 3.29 mg/g, respectively, while those for regenerated and dried CMFs were 22.67% and 0.91 mg/g, respectively, as shown in Figure 7c and Table S4. As the CaCO_3_ concentration of the CMF composite increased, the LE and adsorption capacity increased, indicating that the printed CMF can control the uptake of 5-FU.

The profile of the controlled release of 5-FU was studied using the printed CMF/CaCO_3_ composite to reduce the rapid drug release that has adverse effects on healthy cells. The results of the statistical analysis are simplified in Table S5 and interpreted in Figure 7d. The burst release can be seen for CaCO_3_ (see Figure 7d), where 30% of the drug is released in PBS solution that mimics the small intestine fluid conditions within the first hour. Meanwhile, CMFs have just 7.28% release in their first hour, showing the potential of CMFs to be utilized in drug carrier composite. The initial release can be seen to increase with increasing CaCO_3_ concentration in the CMF printed structure, ranging from 8 to 12% for CMF/0.25CaCO_3_ to CMF/1.00CaCO_3,_ respectively. In the next hours, the drug release steadily increased until the 21st hour, while CaCO_3_ achieved equilibrium after the 13th hour. This is because the porous structure of CaCO_3_ assists in the rapid uptake and release of 5-FU, while the slow disintegration of fibrillated cellulose composited with CaCO_3_ will reduce the release process of 5-FU. The desired more sustained 5-FU release profile that can be observed for printed biocompatible CMF/CaCO_3_ composite can be used for other types of drugs such as doxorubicin [31,61].

## 4. Conclusions

In summary, a remarkable strong and flexible cellulose-based 3D-printed structure with the cellulose fibrils intact was constructed using a partial dissolution of CMFs in an alkaline solvent. The printable CMF solution that possesses shear-thinning properties with high viscosity at a low shear rate desired for DIW 3D printing was able to print complex shapes such as scaffold, logo, and human ear cartilage with high precision and shape fidelity. The printed CMF structure exhibited excellent mechanical properties with tensile strength of 66 MPa, Young’s modulus of 2.16 GPa, and elongation of 8.76%. Interestingly, the retained fibrillated form of CMFs is able to control the uptake and release of a therapeutic drug, 5-FU, by introducing CaCO_3_ as a drug carrier in a 3D-printed CMF/CaCO_3_ composite. The CMF printable composite showed the potential for use in controlled drug delivery applications and inhibits the initial burst drug release that has adverse effects on healthy cells.

## Figures and Tables

**Table 1:** Cellulose I and cellulose II fractions at CMF different concentrations.

Samples	Cellulose I Fraction	Cellulose II Fraction	Amorphous Fraction	Cellulose I/Cellulose II	Crystallinity Index, Crl (%)
CMF	0.607	0.047	0.345	12.795	65.49
CMF 3%	0.037	0.424	0.539	0.088	46.08
CMF 5%	0.246	0.313	0.522	0.786	51.76
CMF 7%	0.328	0.233	0.439	1.407	56.06
CMF 8%	0.374	0.216	0.410	1.734	58.97
CMF 9%	0.420	0.186	0.394	2.254	60.59

## Data Availability

Not applicable.

## Supplementary Materials

The following are available online at https://www.mdpi.com/article/10.3390/polym13121912/s1. Table S1. Volumetric flow rate, Q_v_ vs Extrusion Speed of CMF solution. The data are presented as mean ± standard deviation (n = 3); Table S2. Volumetric flow rate, Q_v_ vs Extrusion Speed of CMF solution. The data are presented as mean ± standard deviation (n = 3); Table S3. Mechanical Properties of 3D printed CMF. The data are presented as mean ± standard deviation (n = 3); Table S4. 5-FU uptake data on CMF/CaCO_3_ composite printed structure. The data are presented as mean ± standard deviation (n = 3); Table S5. One way ANOVA results on kinetic release of 5-FU (%) for 24 h (Tukey Method; N: 3; α = 0.05; Equal variances were assumed for the analysis); Figure S1. Printing of CMF tensile sample according to ASTM D638 Type IV; Figure S2. Tensile testing of regenerated and dried printed CMF tensile sample.
