# Supplementary material for: Intact Fibrillated 3D-Printed Cellulose Macrofibrils/CaCO3 for Controlled Drug Delivery
Source: Polymers (Basel). 2021 Jun 8;13(12):1912. doi: 10.3390/polym13121912 (PMC8227662; doi:10.3390/polym13121912)
Supplement: Supplementary file 1 [file polymers-13-01912-s001.zip › polymers-1244921-supplementary.pdf]

polymers

Supplementary Materials: Intact Fibrillated 3D-Printed Cellulose Macrofibrils/$CaCO_3$ for Controlled Drug Delivery

Denesh Mohan, Zee Khai Teong, Mohd Shaiful Sajab, Nur Hidayatul Nazirah Kamarudin and Hatika Kaco

Table S1. Volumetric flow rate, Q_v vs Extrusion Speed of CMF solution. The data are presented as mean ± standard deviation (n = 3).

Extrusion Speed (mm/s)	Volumetric Flow Rate, Q_v (mm^3/s)
0.4174 ± 0.0125	7.2501 ± 0.2042
0.5146 ± 0.0134	8.5823 ± 0.3251
0.6091 ± 0.0113	9.5833 ± 0.2750
0.6862 ± 0.0206	11.1667 ± 0.3583
0.7855 ± 0.0186	13.0033 ± 0.4267

Table S2. Volumetric flow rate, Q_v vs Extrusion Speed of CMF solution. The data are presented as mean ± standard deviation (n = 3).

Print Speed, v (mm/s)	Print Line Width, x (mm)	Print Line Height, h (mm)	Print Cross-sectional Area, A (mm^2)
5	1.68 ± 0.07	1.39 ± 0.06	1.84 ± 0.12
10	1.24 ± 0.09	0.95 ± 0.03	0.93 ± 0.15
15	0.92 ± 0.06	0.86 ± 0.04	0.62 ± 0.13
20	0.80 ± 0.05	0.78 ± 0.06	0.49 ± 0.12
25	0.71 ± 0.07	0.68 ± 0.04	0.37 ± 0.13
30	0.65 ± 0.09	0.63 ± 0.06	0.32 ± 0.14

Table S3. Mechanical Properties of 3D printed CMF. The data are presented as mean ± standard deviation (n = 3).

Samples	Tensile Strength (MPa)	Young's Modulus (GPa)	Strain %)
CMF 7%	48.96 ± 1.02	1.59 ± 0.06	7.54 ± 0.12
CMF 8%	58.89 ± 1.36	1.91 ± 0.07	8.05 ± 0.08
CMF 9%	66.70 ± 0.98	2.16 ± 0.06	8.76 ± 0.09

Table S4. 5-FU uptake data on CMF/$CaCO_3$ composite printed structure. The data are presented as mean ± standard deviation (n = 3).

Samples	C_0 (ppm)	C_e (ppm)	LE (%)	q_e (mg/g)
CMF	8	6.19	22.67 ± 0.53	0.91 ± 0.03
CMF:0.25$CaCO_3$		5.73	28.36 ± 0.41	1.13 ± 0.05
CMF:0.50$CaCO_3$		5.35	33.17 ± 0.62	1.33 ± 0.05
CMF:0.75$CaCO_3$		4.87	39.13 ± 0.56	1.57 ± 0.06
CMF:1.00$CaCO_3$		4.45	44.41 ± 0.47	1.78 ± 0.04
$CaCO_3$		1.40	82.49 ± 0.51	3.29 ± 0.07

C_0 and C_e, initial and equilibrium concentrations of the 5-FU (mg/L); LE, loading efficiency of 5-FU at final equilibrium; q_e, total uptake amount of 5-FU at final equilibrium.

Table S5. One way ANOVA results on kinetic release of 5-FU (%) for 24 h (Tukey Method; N: 3; α = 0.05; Equal variances were assumed for the analysis)

Samples	Time (h)	Mean	StDev	Grouping
$CaCO_3$	1	29.493	0.300	A
CMF:1.00$CaCO_3$		11.132	0.195	B
CMF:0.75$CaCO_3$		9.687	0.230	C
CMF:0.50$CaCO_3$		8.857	0.241	D
CMF:0.25$CaCO_3$		7.345	0.115	E
CMF		7.286	0.100	E
$CaCO_3$	2	48.941	0.201	A
CMF:1.00$CaCO_3$		14.468	0.331	B
CMF:0.75$CaCO_3$		12.454	0.250	C
CMF:0.50$CaCO_3$		11.187	0.248	D
CMF:0.25$CaCO_3$		10.185	0.209	E
CMF		9.314	0.187	F
$CaCO_3$	3	59.610	0.262	A
CMF:1.00$CaCO_3$		18.157	0.431	B
CMF:0.75$CaCO_3$		16.289	0.326	C
CMF:0.50$CaCO_3$		14.257	0.348	D
CMF:0.25$CaCO_3$		13.331	0.300	E
CMF		12.398	0.180	F
$CaCO_3$	4	67.096	0.202	A
CMF:1.00$CaCO_3$		22.081	0.431	B
CMF:0.75$CaCO_3$		19.083	0.382	C
CMF:0.50$CaCO_3$		17.456	0.379	D
CMF:0.25$CaCO_3$		16.055	0.322	E
CMF		15.191	0.220	E
$CaCO_3$	5	75.943	0.221	A
CMF:1.00$CaCO_3$		24.931	0.489	B
CMF:0.75$CaCO_3$		22.685	0.287	C
CMF:0.50$CaCO_3$		21.022	0.384	D
CMF:0.25$CaCO_3$		19.648	0.274	E
CMF		19.015	0.276	E
$CaCO_3$	6	82.122	0.295	A
CMF:1.00$CaCO_3$		30.764	0.188	B
CMF:0.75$CaCO_3$		26.213	0.382	C
CMF:0.50$CaCO_3$		22.888	0.430	D
CMF:0.25$CaCO_3$		21.109	0.295	E
CMF		19.929	0.289	F
$CaCO_3$	7	85.992	0.280	A
CMF:1.00$CaCO_3$		34.391	0.214	B
CMF:0.75$CaCO_3$		30.491	0.140	C
CMF:0.50$CaCO_3$		24.269	0.283	D
CMF:0.25$CaCO_3$		22.570	0.315	E
CMF		20.386	0.296	F

$CaCO_3$	8	87.579	0.340	A					
CMF:1.00$CaCO_3$		38.623	0.245		B				
CMF:0.75$CaCO_3$		33.598	0.154			C			
CMF:0.50$CaCO_3$		27.465	0.333				D		
CMF:0.25$CaCO_3$		24.793	0.278					E	
CMF		21.152	0.256						F
$CaCO_3$	9	88.556	0.350	A					
CMF:1.00$CaCO_3$		40.995	0.262		B				
CMF:0.75$CaCO_3$		36.758	0.169			C			
CMF:0.50$CaCO_3$		30.592	0.262				D		
CMF:0.25$CaCO_3$		27.200	0.305					E	
CMF		22.214	0.322						F
$CaCO_3$	10	88.742	0.430	A					
CMF:1.00$CaCO_3$		44.717	0.290		B				
CMF:0.75$CaCO_3$		39.339	0.268			C			
CMF:0.50$CaCO_3$		32.404	0.282				D		
CMF:0.25$CaCO_3$		28.366	0.318					E	
CMF		22.763	0.330						F
$CaCO_3$	11	88.887	0.315	A					
CMF:1.00$CaCO_3$		47.461	0.311		B				
CMF:0.75$CaCO_3$		40.916	0.279			C			
CMF:0.50$CaCO_3$		34.905	0.310				D		
CMF:0.25$CaCO_3$		30.554	0.343					E	
CMF		23.677	0.343						F
$CaCO_3$	12	88.926	0.258	A					
CMF:1.00$CaCO_3$		49.709	0.445		B				
CMF:0.75$CaCO_3$		45.184	0.207			C			
CMF:0.50$CaCO_3$		37.468	0.340				D		
CMF:0.25$CaCO_3$		32.085	0.360					E	
CMF		24.285	0.275						F
$CaCO_3$	13	88.9741	0.118	A					
CMF:1.00$CaCO_3$		52.438	0.349		B				
CMF:0.75$CaCO_3$		47.385	0.357			C			
CMF:0.50$CaCO_3$		40.481	0.289				D		
CMF:0.25$CaCO_3$		32.989	0.299					E	
CMF		25.437	0.283						F
$CaCO_3$	14	89.0158	0.135	A					
CMF:1.00$CaCO_3$		55.974	0.377		B				
CMF:0.75$CaCO_3$		50.007	0.202			C			
CMF:0.50$CaCO_3$		42.354	0.306				D		
CMF:0.25$CaCO_3$		34.883	0.316					E	
CMF		25.811	0.275						F

$CaCO_3$	15	89.062	0.167	A					
CMF:1.00$CaCO_3$		58.166	0.386		B				
CMF:0.75$CaCO_3$		52.186	0.325			C			
CMF:0.50$CaCO_3$		45.413	0.335				D		
CMF:0.25$CaCO_3$		36.048	0.327					E	
CMF		26.084	0.278						F
$CaCO_3$	16	89.092	0.240	A					
CMF:1.00$CaCO_3$		60.068	0.409		B				
CMF:0.75$CaCO_3$		54.754	0.221			C			
CMF:0.50$CaCO_3$		48.534	0.257				D		
CMF:0.25$CaCO_3$		37.213	0.337					E	
CMF		26.358	0.281						F
$CaCO_3$	17	89.112	0.230	A					
CMF:1.00$CaCO_3$		63.324	0.435		B				
CMF:0.75$CaCO_3$		56.846	0.256			C			
CMF:0.50$CaCO_3$		51.089	0.274				D		
CMF:0.25$CaCO_3$		39.325	0.356					E	
CMF		26.905	0.287						F
$CaCO_3$	18	89.131	0.205	A					
CMF:1.00$CaCO_3$		67.112	0.332		B				
CMF:0.75$CaCO_3$		58.427	0.263			C			
CMF:0.50$CaCO_3$		52.897	0.286				D		
CMF:0.25$CaCO_3$		41.409	0.332					E	
CMF		27.270	0.291						F
$CaCO_3$	19	89.132	0.212	A					
CMF:1.00$CaCO_3$		70.210	0.490		B				
CMF:0.75$CaCO_3$		61.064	0.275			C			
CMF:0.50$CaCO_3$		54.705	0.298				D		
CMF:0.25$CaCO_3$		43.112	0.391					E	
CMF		27.562	0.271						F
$CaCO_3$	20	89.159	0.226	A					
CMF:1.00$CaCO_3$		72.676	0.510		B				
CMF:0.75$CaCO_3$		62.667	0.440			C			
CMF:0.50$CaCO_3$		55.453	0.303				D		
CMF:0.25$CaCO_3$		45.078	0.408					E	
CMF		27.918	0.286						F
$CaCO_3$	21	89.156	0.240	A					
CMF:1.00$CaCO_3$		74.196	0.376		B				
CMF:0.75$CaCO_3$		63.131	0.222			C			
CMF:0.50$CaCO_3$		56.201	0.308				D		
CMF:0.25$CaCO_3$		47.216	0.354					E	
CMF		28.091	0.300						F

$CaCO_3$	22	89.162	0.193	A					
CMF:1.00$CaCO_3$		74.956	0.528		B				
CMF:0.75$CaCO_3$		64.228	0.290			C			
CMF:0.50$CaCO_3$		57.270	0.316				D		
CMF:0.25$CaCO_3$		48.064	0.436					E	
CMF		28.201	0.277						F
$CaCO_3$	23	89.162	0.222	A					
CMF:1.00$CaCO_3$		75.701	0.534		B				
CMF:0.75$CaCO_3$		64.926	0.396			C			
CMF:0.50$CaCO_3$		57.276	0.316				D		
CMF:0.25$CaCO_3$		48.218	0.338					E	
CMF		28.273	0.302						F
$CaCO_3$	24	89.172	0.229	A					
CMF:1.00$CaCO_3$		75.705	0.534		B				
CMF:0.75$CaCO_3$		64.914	0.293			C			
CMF:0.50$CaCO_3$		57.281	0.316				D		
CMF:0.25$CaCO_3$		48.379	0.321					E	
CMF		28.393	0.267						F

*Means that do not share a letter are significantly different; $p < 0.05$

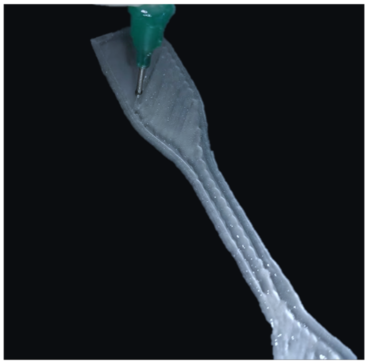

Figure S1. Printing of CMF tensile sample according to ASTM D638 Type IV.

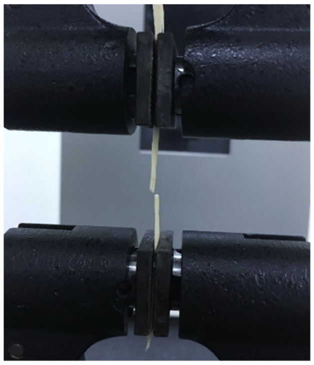

Figure S2. Tensile testing of regenerated and dried printed CMF tensile sample.

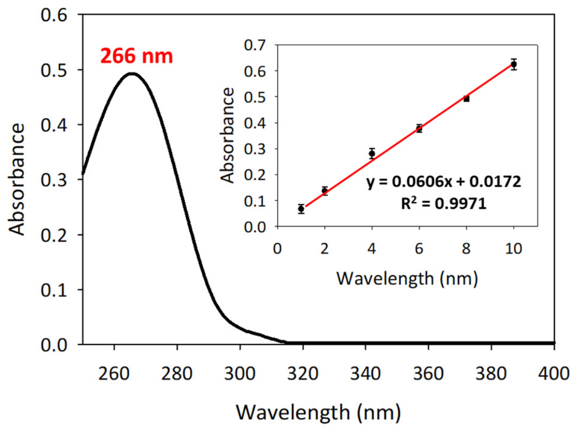

Figure S3. (a) UV Spectrum of 5-fluorouracil (5-FU) (inset shows linear calibration curve according to the Beer-Lambert law). The data are presented as mean ± standard deviation (n = 3).
